# Supplementary material for: Integrating UHPLC-MS and MALDI-MSI for Spatial Nucleoside Profiling in FFPE Breast Cancer: A Multimodal Molecular Pathology Framework
Source: Anal Chem. 2026 Sep 17;98(38):27906–17. doi: 10.1021/acs.analchem.6c00978 (PMC13629170; doi:10.1021/acs.analchem.6c00978)
Supplement: Supplementary file 1 [file ac6c00978_si_001.pdf]

Supporting information:

Integrating UHPLC-MS and MALDI-MSI for Spatial Nucleoside Profiling in FFPE Breast Cancer: A Multimodal Molecular Pathology Framework

Jierong Wu[1], Sabrina Y. Geisberger[1,5], Guido Mastrobuoni[1,5], Kamil Lisek[2], Sandra Raimundo[2], Grit Nebrich[3,6], Marta Grzeski[3], Nikolaus Rajewsky[2,7,8,9,10,11], Frederick Klauschen[4], Oliver Klein[3,6*], Stefan Kempa[1,5*]

1 Integrative Proteomics and Metabolomics, Berlin Institute for Medical Systems Biology (BIMSB), Max Delbrück Center for Molecular Medicine in the Helmholtz Association (MDC), 13125 Berlin, Germany.

2 Systems Biology of Gene Regulatory Elements, Berlin Institute for Medical Systems Biology (BIMSB), Max Delbrück Center for Molecular Medicine in the Helmholtz Association (MDC), 13125 Berlin, Germany.

3 BIH Center for Regenerative Therapies (BCRT), Berlin Institute of Health at Charité – Universitätsmedizin Berlin, 13353 Berlin, Germany.

4 Institute of Pathology, Ludwig Maximilians University Hospital Munich, 80336 Munich, Germany.

5 MDC–Bruker Center of Excellence for Single Cell OMICS, 10115 Berlin, Germany.

6 Imaging Mass Spectrometry Unit, Berlin Institute of Health at Charité – Universitätsmedizin Berlin, 13353 Berlin, Germany.

7 Charité – Universitätsmedizin Berlin, corporate member of Freie Universität Berlin and Humboldt-Universität zu Berlin, 10117 Berlin, Germany.

8 NeuroCure Cluster of Excellence, Charité – Universitätsmedizin Berlin, 10117 Berlin, Germany.

9 German Center for Cardiovascular Research (DZHK), Partner Site Berlin, 10117 Berlin, Germany.

10 German Cancer Consortium (DKTK), 69120 Heidelberg, Germany.

11 National Center for Tumor Diseases (NCT), Partner Site Berlin, 10117 Berlin, Germany.

*Corresponding authors:

Stefan Kempa

Email: stefan.kempa@mdc-berlin.de

Oliver Klein

Email: oliver.klein@bih-charite.de

Contents

Figure S1. Sample preparation and analysis workflow. 3

Figure S2. Heatmap showing relative nucleoside abundance across individual biological replicates. 4

Figure S3. Spatial distribution of nucleosides in breast tissue samples at different tumor stages. 7

Figure S4. ROC analysis of nucleoside signals for tumor stage classification. 9

Figure S5. Box plots illustrating the normalized signal intensities of nucleosides detected by MALDI-MSI across different stages of tumor progression. 10

Figure S6. Overview of spatial distribution of nucleosides in FFPE clinical breast cancer tissues. 12

Figure S7. Multimodal visualization of deoxycytidine distribution and phenotypic tumor markers in FFPE murine breast tumors. 13

Table S1. UHPLC–MS/MS acquisition parameters for nucleoside analysis. 14

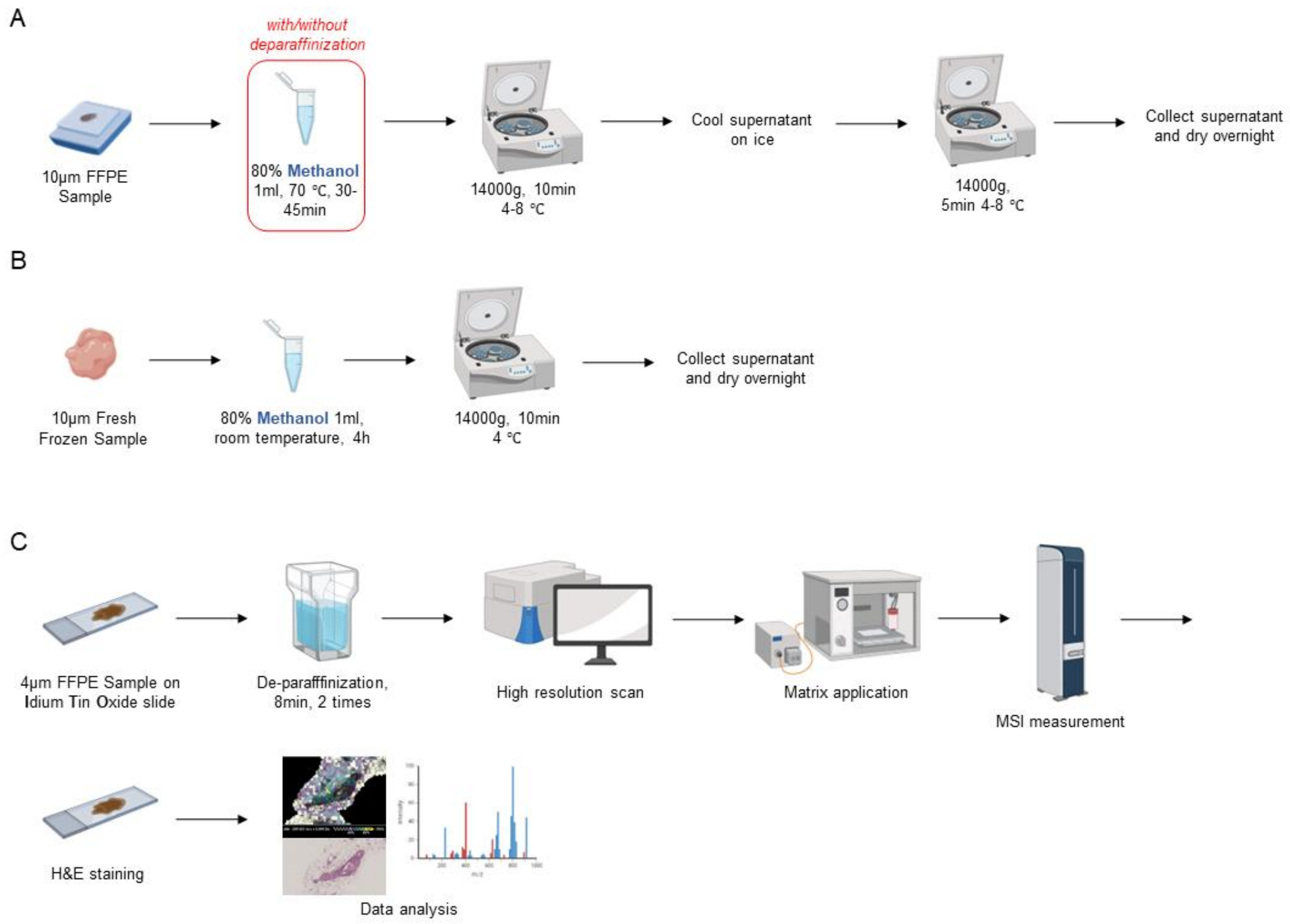

Figure S1. Sample preparation and analysis workflow.

(A) Workflow for nucleoside extraction from FFPE tissue sections (10 µm), with or without xylene-based deparaffinization.

(B) Workflow for fresh-frozen tissue extraction.

(C) FFPE tissue sections (4 µm) on ITO-coated slides underwent deparaffinization, matrix application, and MALDI-MSI analysis, followed by H&E staining and data processing for spatial metabolite distribution analysis.

A

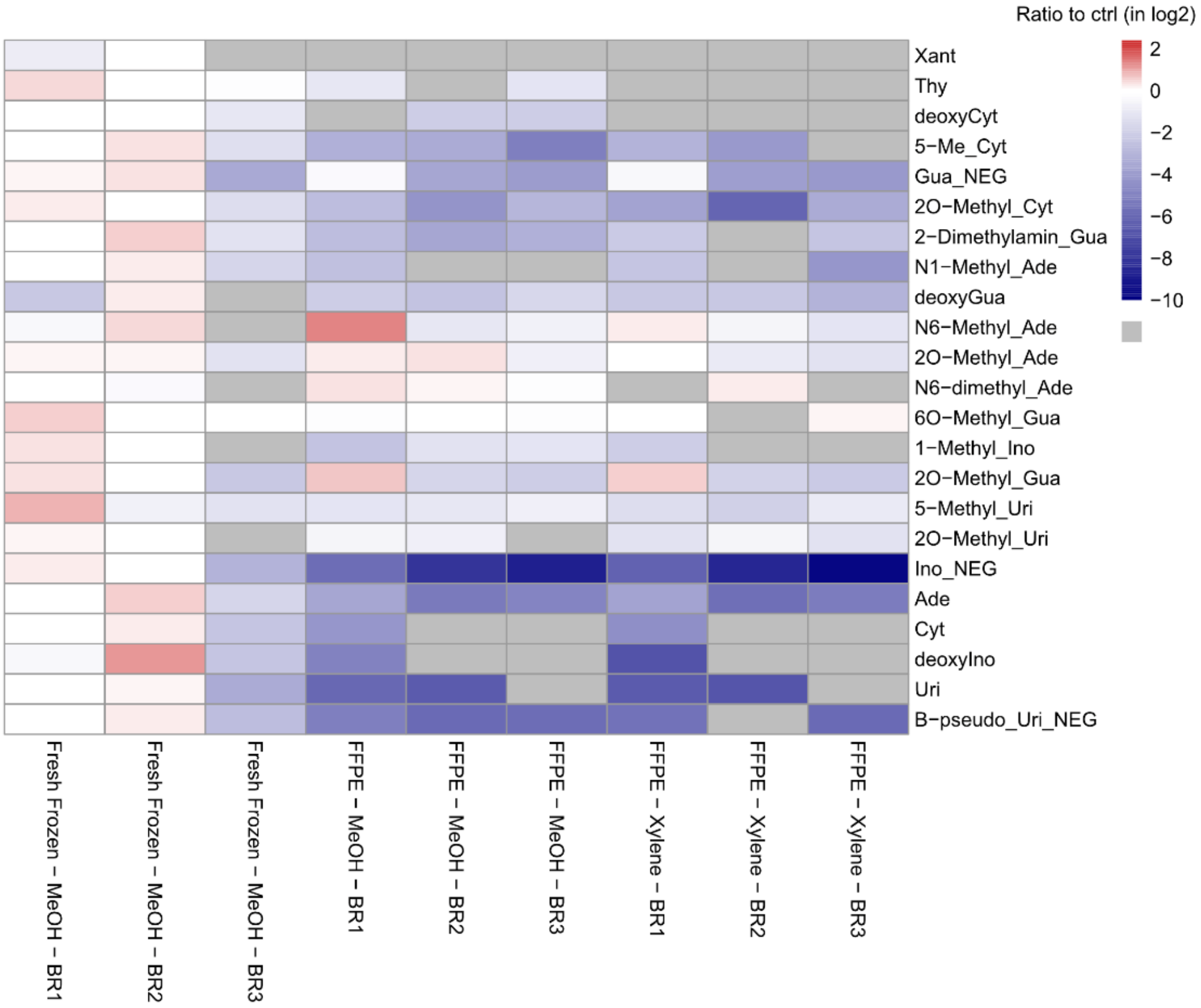
Ratio to ctrl (in log2)
2
0
−2
−4
−6
−8
−10
Xant
Thy
deoxyCyt
5−Me_Cyt
Gua_NEG
2O−Methyl_Cyt
2−Dimethylamin_Gua
N1−Methyl_Ade
deoxyGua
N6−Methyl_Ade
2O−Methyl_Ade
N6−dimethyl_Ade
6O−Methyl_Gua
1−Methyl_Ino
2O−Methyl_Gua
5−Methyl_Uri
2O−Methyl_Uri
Ino_NEG
Ade
Cyt
deoxyIno
Uri
B−pseudo_Uri_NEG
Fresh Frozen − MeOH − BR1
Fresh Frozen − MeOH − BR2
Fresh Frozen − MeOH − BR3
FFPE − MeOH − BR1
FFPE − MeOH − BR2
FFPE − MeOH − BR3
FFPE − Xylene − BR1
FFPE − Xylene − BR2
FFPE − Xylene − BR3

B

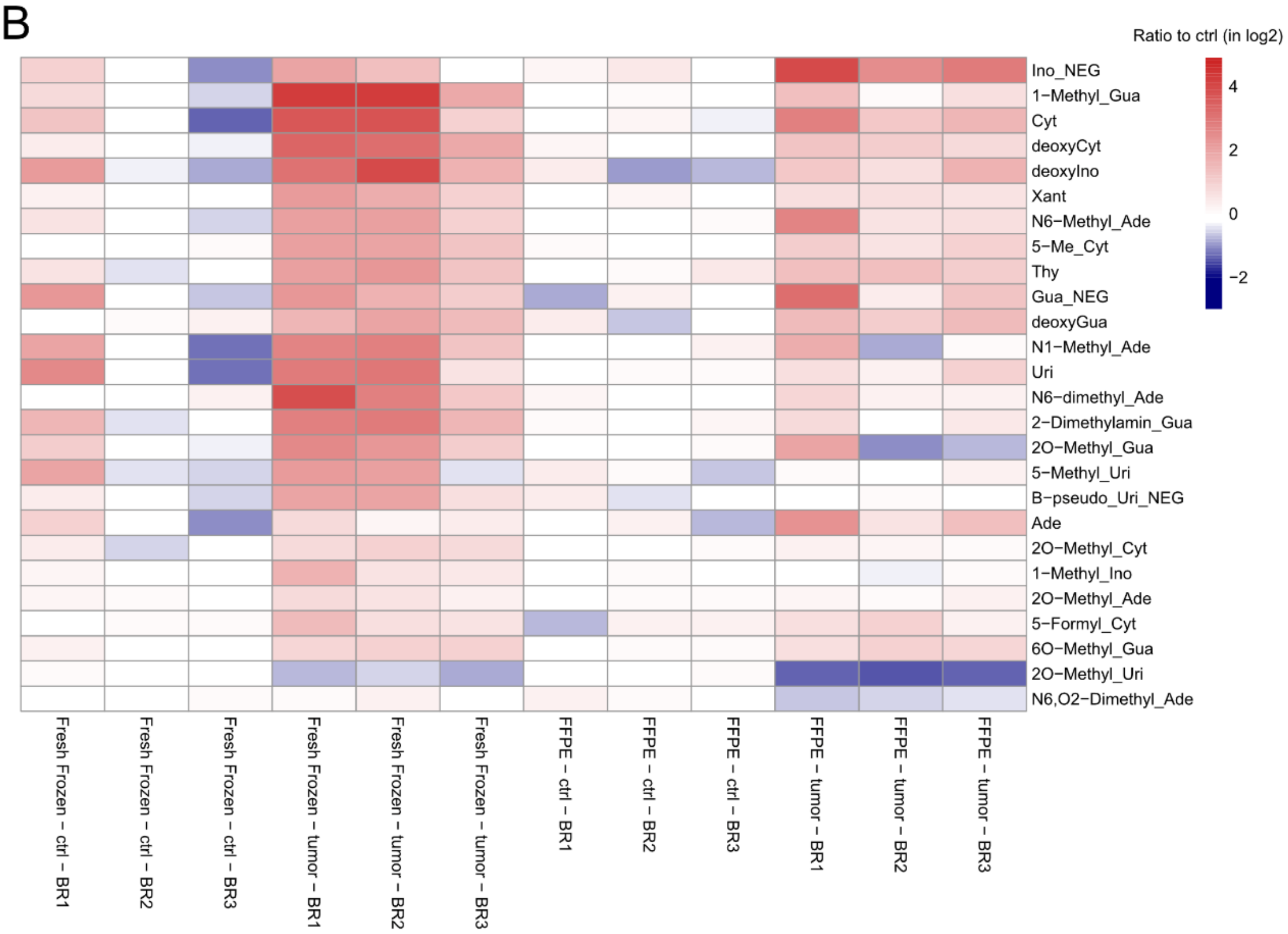
Ratio to ctrl (in log2)
4
2
0
−2
Ino_NEG
1−Methyl_Gua
Cyt
deoxyCyt
deoxyIno
Xant
N6−Methyl_Ade
5−Me_Cyt
Thy
Gua_NEG
deoxyGua
N1−Methyl_Ade
Uri
N6−dimethyl_Ade
2−Dimethylamin_Gua
2O−Methyl_Gua
5−Methyl_Uri
B−pseudo_Uri_NEG
Ade
2O−Methyl_Cyt
1−Methyl_Ino
2O−Methyl_Ade
5−Formyl_Cyt
6O−Methyl_Gua
2O−Methyl_Uri
N6,O2−Dimethyl_Ade
Fresh Frozen − ctrl − BR1
Fresh Frozen − ctrl − BR2
Fresh Frozen − ctrl − BR3
Fresh Frozen − tumor − BR1
Fresh Frozen − tumor − BR2
Fresh Frozen − tumor − BR3
FFPE − ctrl − BR1
FFPE − ctrl − BR2
FFPE − ctrl − BR3
FFPE − tumor − BR1
FFPE − tumor − BR2
FFPE − tumor − BR3

Figure S2. Heatmaps showing relative nucleoside abundance across individual biological replicates.

The heatmaps display log2-transformed ratios of nucleoside signals relative to the control group. Rows represent detected nucleosides and modified nucleosides, while columns represent individual biological replicates from FFPE and fresh-frozen tissue samples, extracted with methanol (MeOH) or Xylene (A) and control (ctrl) or tumor tissue (B). Red indicates increased abundance relative to control, blue indicates decreased abundance, and white indicates little or no change. This representation shows the biological variability among individual samples.

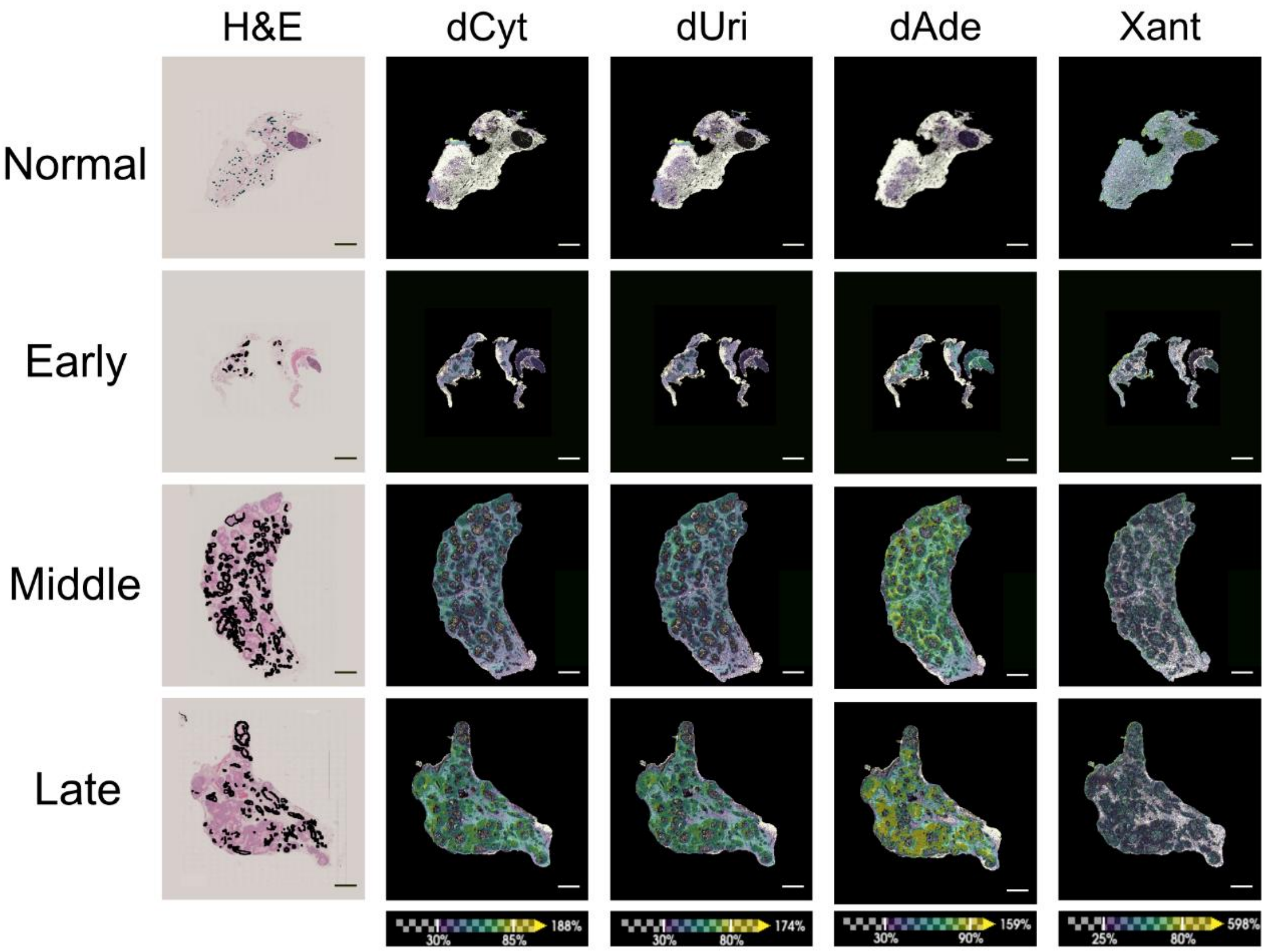
H&E
dCyt
dUri
dAde
Xant
Normal
Early
Middle
Late
30% 85% 188%
30% 80% 174%
30% 90% 159%
25% 80% 598%

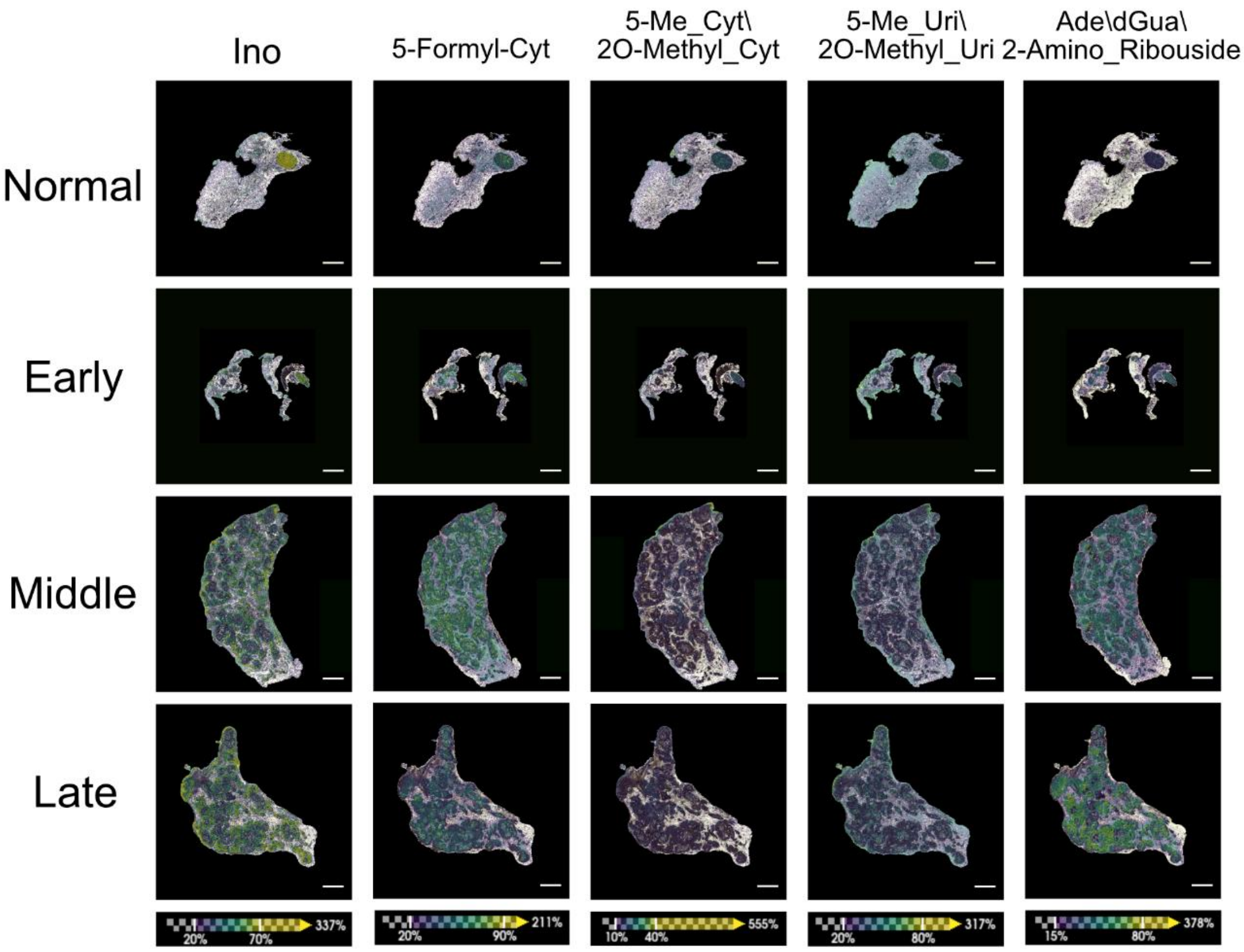
Ino
5-Formyl-Cyt
5-Me_Cyt\
2O-Methyl_Cyt
5-Me_Uri\
2O-Methyl_Uri
Ade\dGua\
2-Amino_Ribouside
Normal
Early
Middle
Late
20% 70% 337%
20% 90% 211%
10% 40% 555%
20% 80% 317%
15% 80% 378%

Figure S3. Spatial distribution of nucleosides in breast tissue samples at different tumor stages.

Representative MALDI-MSI ion images of 9 nucleosides detected in FFPE breast tissues across normal, early, mid, and late tumor stages. Corresponding H&E-stained images are shown for histological reference. Scale bars: 2 mm.

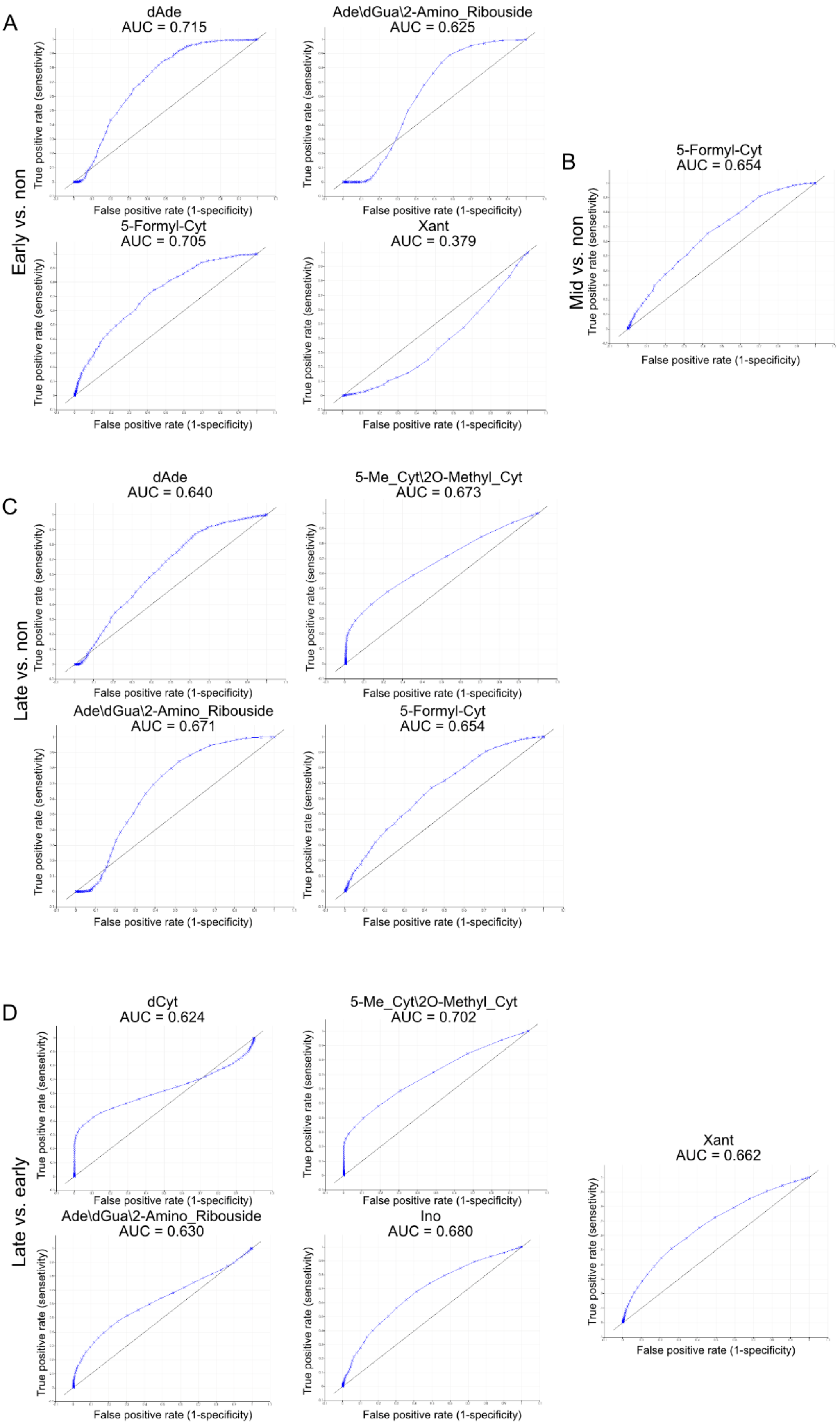
A
Early vs. non
dAde
AUC = 0.715
Ade\dGua\2-Amino_Ribouside
AUC = 0.625
5-Formyl-Cyt
AUC = 0.705
Xant
AUC = 0.379
B
Mid vs. non
5-Formyl-Cyt
AUC = 0.654
C
Late vs. non
dAde
AUC = 0.640
5-Me_Cyt\2O-Methyl_Cyt
AUC = 0.673
Ade\dGua\2-Amino_Ribouside
AUC = 0.671
5-Formyl-Cyt
AUC = 0.654
D
Late vs. early
dCyt
AUC = 0.624
5-Me_Cyt\2O-Methyl_Cyt
AUC = 0.702
Ade\dGua\2-Amino_Ribouside
AUC = 0.630
Ino
AUC = 0.680
Xant
AUC = 0.662
True positive rate (sensetivity)
False positive rate (1-specificity)

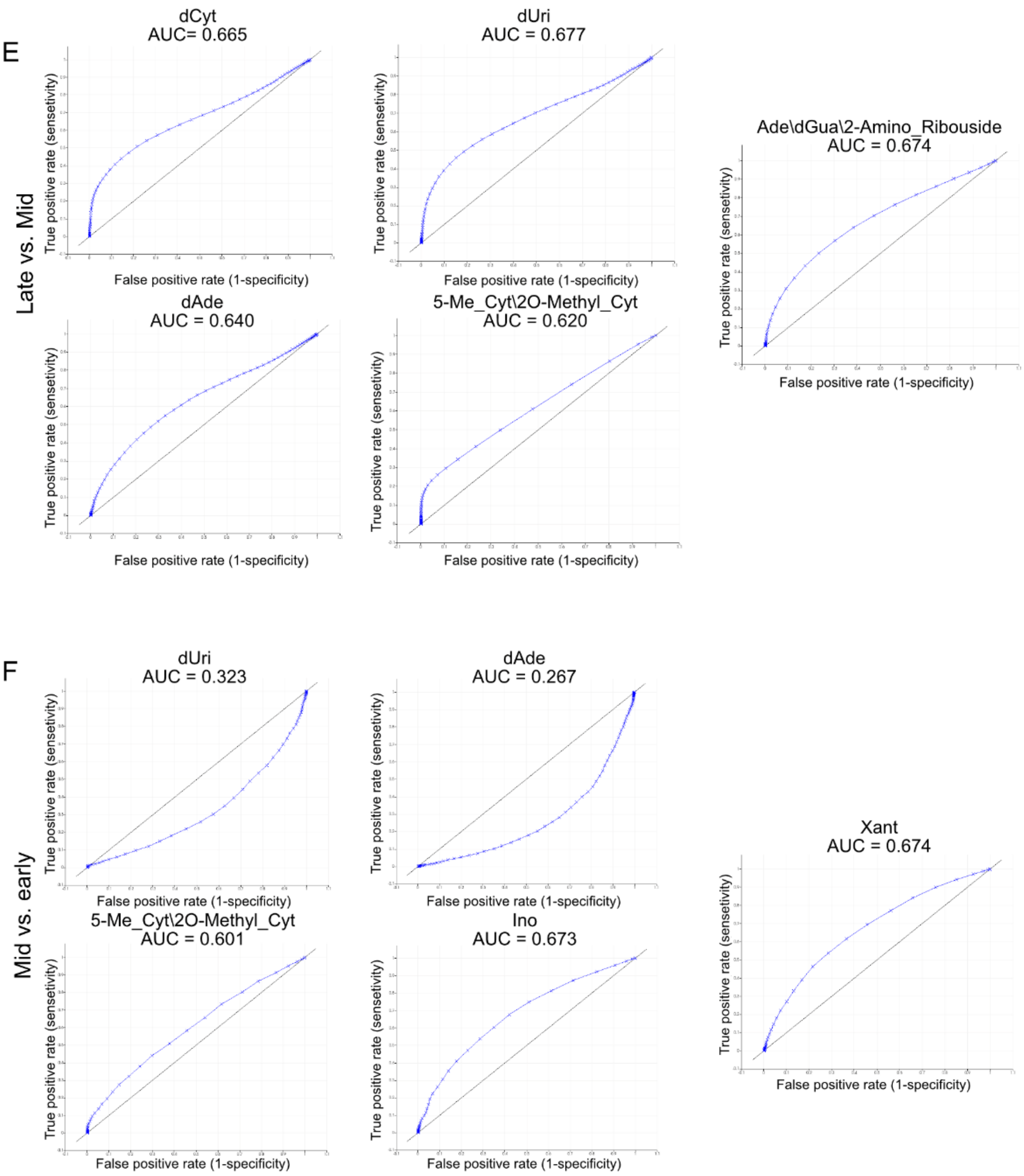

Figure S4. ROC analysis of nucleoside signals for tumor stage classification.

Receiver operating characteristic (ROC) curves for individual nucleosides based on MALDI-MSI intensity data. The area under the curve (AUC) is indicated for each comparison. (A) Early-stage tumors vs. normal tissue; (B) Mid-stage tumors vs. normal tissue; (C) Late-stage tumors vs. normal tissue; (D) Late-stage tumors vs. early-stage tumors; (E) Late-stage tumors vs. mid-stage tumors; (F) Mid-stage tumors vs. early-stage tumors.

Nucleosides with AUC values greater than 0.7 or less than 0.3 were considered to have potential as stage-specific biomarkers.

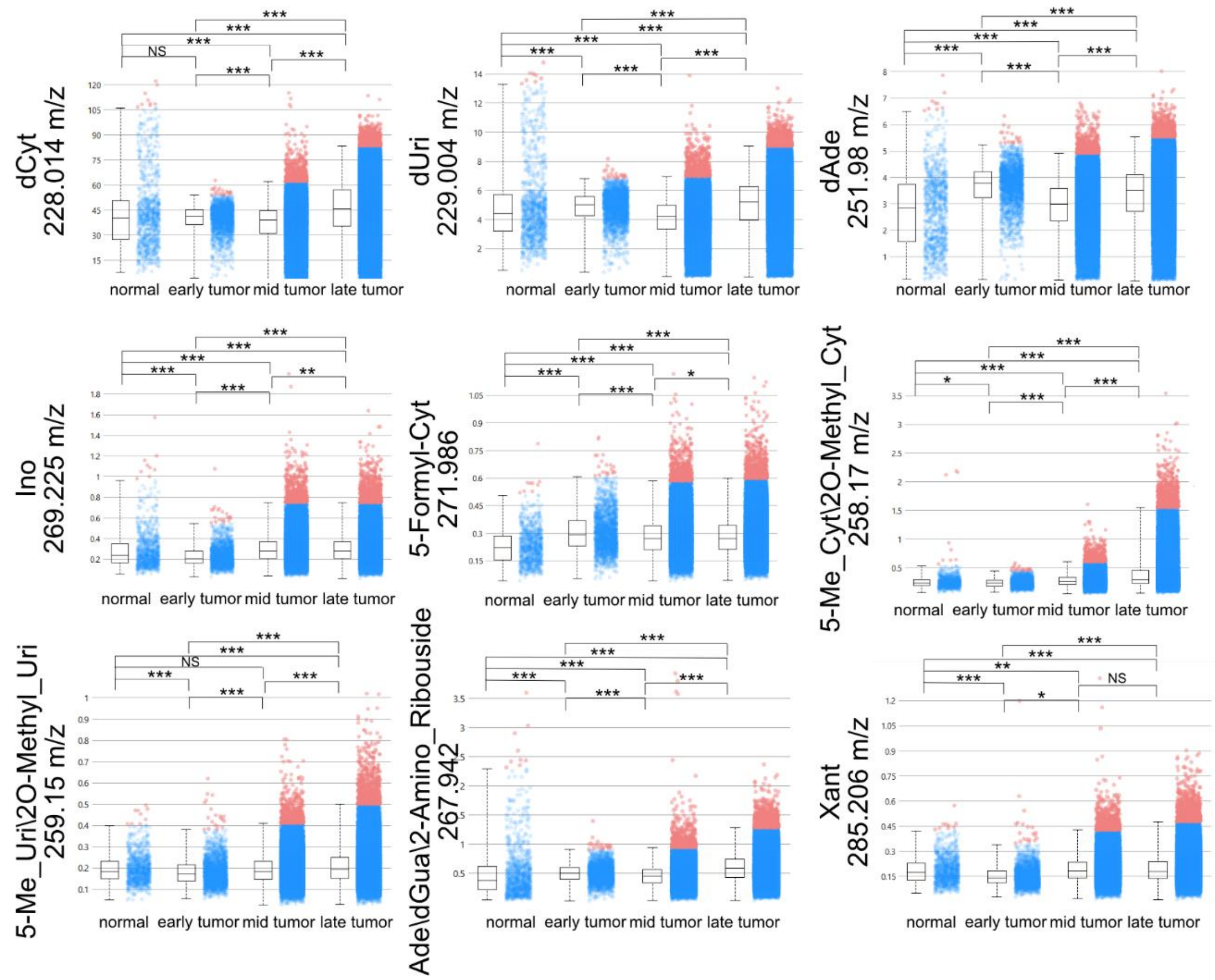

Figure S5. Box plots illustrating the normalized signal intensities of nucleosides detected by MALDI-MSI across different stages of tumor progression.

Samples were categorized into normal, early-stage tumor, mid-stage tumor, and late-stage tumor groups. Each plot displays the distribution of nucleoside intensities across tissue regions, with blue points representing individual pixel measurements and red points denoting statistical outliers. Statistical comparisons between groups were performed using Wilcoxon Rank Sum. Significance levels are indicated as follows: $p < 0.05$ (*), $p < 0.01$ (**), $p < 0.001$ (***), and ns = not significant.

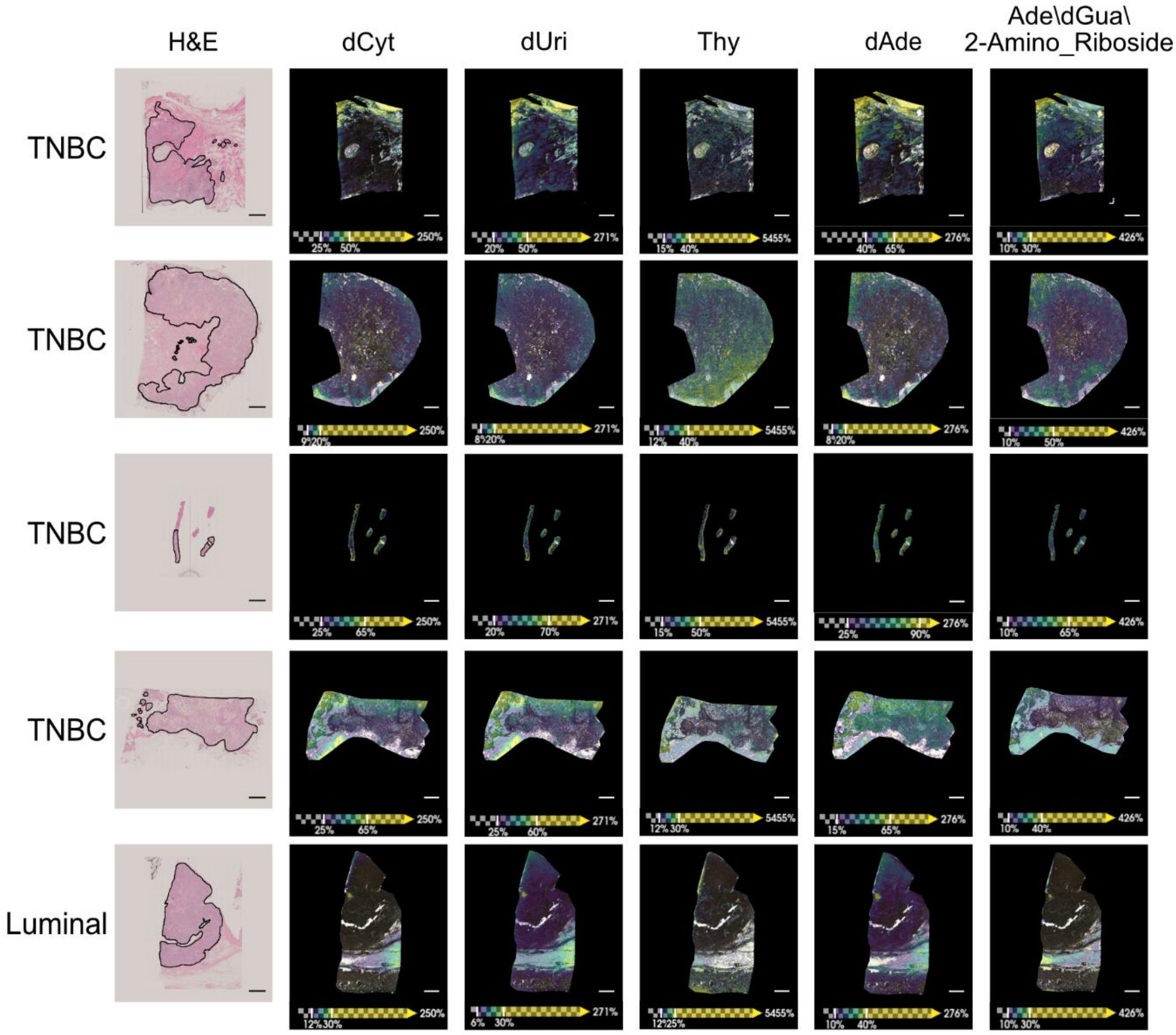
H&E
dCyt
dUri
Thy
dAde
Ade\dGua\
2-Amino_Riboside
TNBC
TNBC
TNBC
TNBC
Luminal

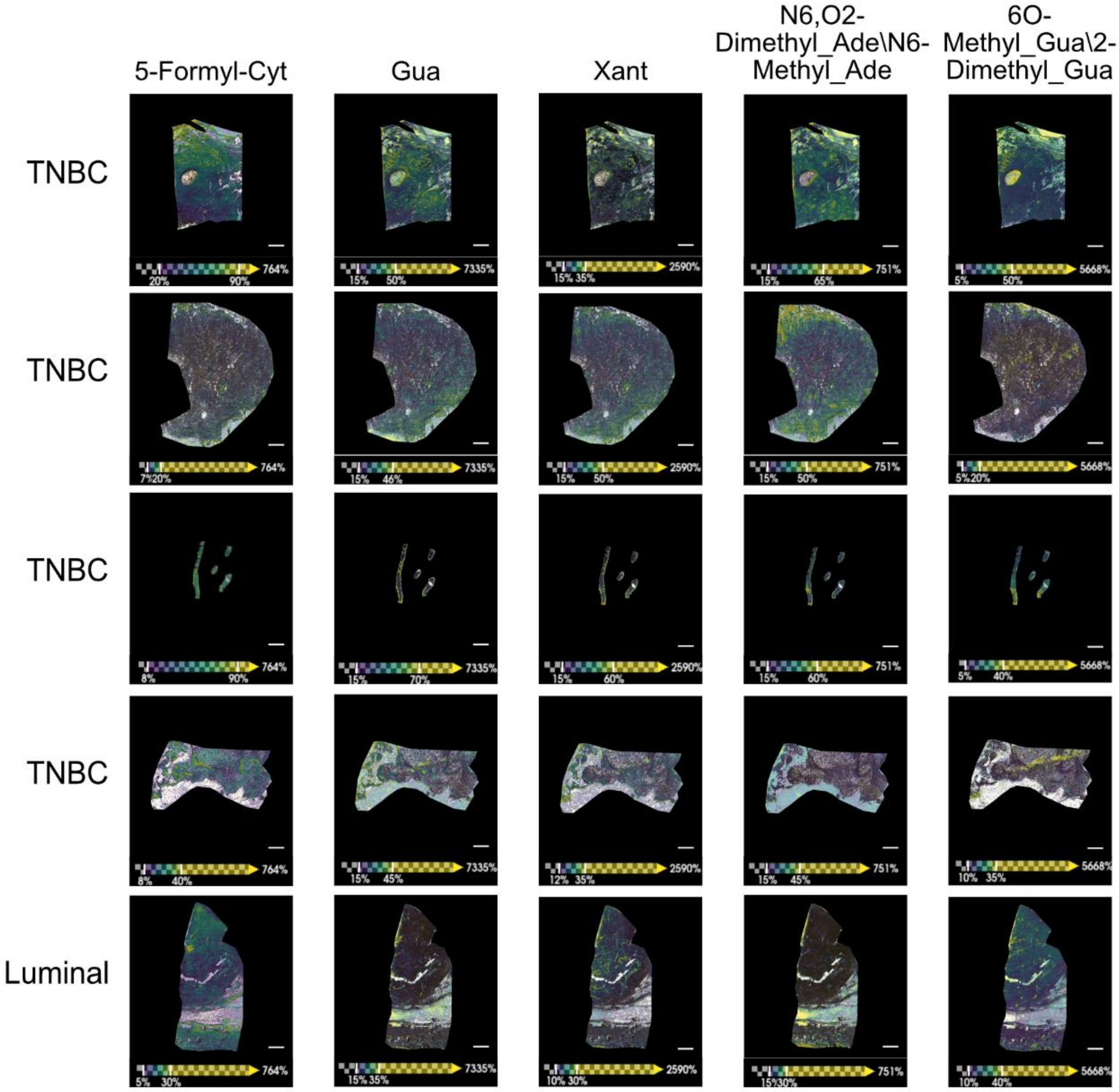

Figure S6. Overview of spatial distribution of nucleosides in FFPE clinical breast cancer tissues.

MALDI-MSI ion images of 10 nucleosides detected in FFPE tissue sections from five breast cancer samples derived from four patients (four TNBC and one luminal subtype). All ion images were normalized to total ion current (TIC) and displayed on a common intensity scale. Scale bars: 2 mm.

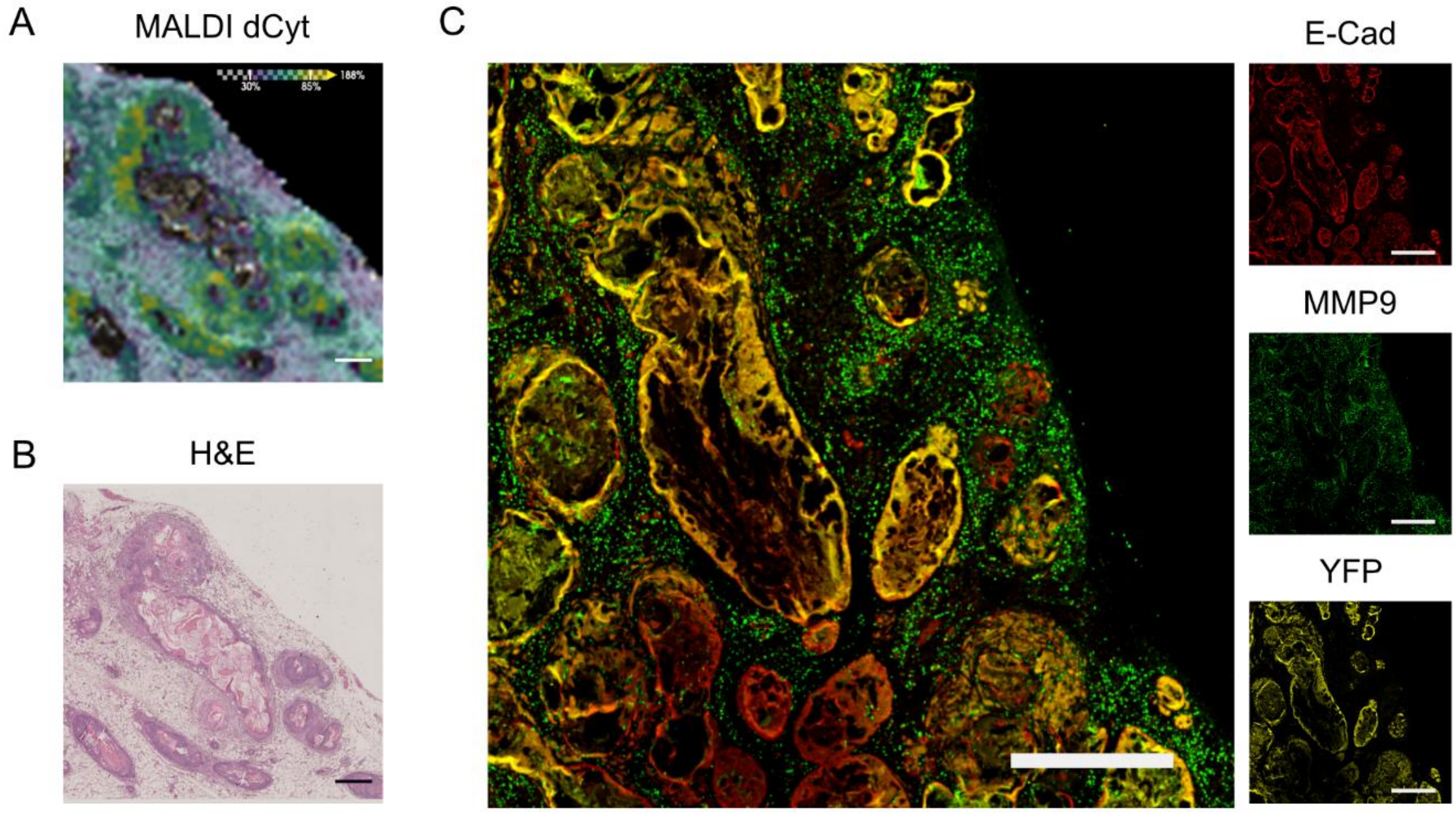

Figure S7. Multimodal visualization of deoxycytidine distribution and phenotypic tumor markers in FFPE murine breast tumors.

(A) MALDI-MSI ion image showing the spatial distribution of deoxycytidine (dCyt) in a representative FFPE breast tumor section from a transgenic murine model, together with the corresponding H&E stain of the same section after matrix removal. Scale bars: 400 µm.

(B) Multiplex immunofluorescence image from a tumor section obtained from a different mouse of the same transgenic model, stained for E-cadherin (red), MMP9 (green), and YFP (yellow), with individual channels shown on the right. These markers highlight epithelial structures (E-cadherin^{+}), proteolytically active tumor/stromal regions (MMP9^{+}), and YFP-positive tumor cells, respectively. Scale bars: 1 mm.

MALDI-MSI and immunofluorescence were thus acquired from different sections of tumors arising in the same mouse model; the observed heterogeneity in marker expression and morphology is consistent with the spatial variation in nucleoside signals revealed by MSI.

Table S1. UHPLC–MS/MS acquisition parameters for nucleoside analysis.

Compound	Polarity	Precursor (m/z)	Product (m/z)	Collision Energy (V)
Deoxy-Cytidine	Positive	228.1	112.1	13
Deoxy-Uridine	Positive	229.1	113.1	14
Thymidine	Positive	243.1	127.1	11
Cytidine	Positive	244.1	112.1	10
Beta-Pseudouridine	Positive	245.07	125.103	15
Uridine	Positive	245.1	113.1	10
dAdenosine	Positive	252.1	136.1	17
5-Methyl-Cytidine	Positive	258.1	126.1	14
2O-Methyl-Cytidine	Positive	258.12	112.1	10
5-Methyl-Uridine	Positive	259.09	127.09	12
2O-Methyl-Uridine	Positive	259.095	113.09	12
Adenosine	Positive	268.1	136.1	18
Deoxy-Guanosine	Positive	268.1	152.1	14
2-Aminopurine Riboside	Positive	268.105	136.105	23
Inosine	Positive	269.083	137.083	14
5-Formyl-Cytidine	Positive	272.083	140.083	12
5-Hydroxymethyl-Cytidine	Positive	274.103	142.103	12
N6-Methyl-Adenosine	Positive	282.11	150.11	19
N1- Methyl-Adenosine	Positive	282.111	150.11	18
2O-Methyl-Adenosine	Positive	282.115	136.11	17
1-Methyl-Inosine	Positive	283.105	151.1	13
Guanosine	Positive	284.1	152.1	13
Xantosine	Positive	285.1	153	12
N6, O2-Dimethyl-Adenosine	Positive	296.133	150.133	18
N6, N6-dimethyl-Adenosine	Positive	296.14	164.14	20
7-Methyl-Guanosine	Positive	298.11	166.11	20
2O-Methyl-Guanosine	Positive	298.115	152.11	14
6-O-Methyl-Guanosine	Positive	298.115	166.115	20
2-(Dimethylamino)guanosine	Positive	312.122	180.122	15
Beta-Pseudourine	Negative	243	153.1	12
Deoxy-Inosine	Negative	251.1	135.1	14
Inosine	Negative	267.1	135.1	15
Guanosine	Negative	282.1	150.1	13

Multiple reaction monitoring (MRM) transitions (precursor/product m/z), ionization polarity, collision energy, and dwell time used for UHPLC–MS/MS detection of nucleosides.
